# Recent Advances in Photoacoustic Imaging: Current Status and Future Perspectives

**DOI:** 10.3390/mi15081007

**Published:** 2024-08-04

**Authors:** Huibin Liu, Xiangyu Teng, Shuxuan Yu, Wenguang Yang, Tiantian Kong, Tangying Liu

**Affiliations:** 1 School of Electromechanical and Automotive Engineering, Yantai University, Yantai 264005, China; cactusil@163.com (H.L.); 15166828596@163.com (X.T.); ysx17753172676@163.com (S.Y.); yangwenguang@ytu.edu.cn (W.Y.); 2 Shandong City Service Institute, Yantai 264005, China

**Keywords:** photoacoustic imaging, photoacoustic microscopy, noncontact photoacoustic, imaging diagnosis

## Abstract

Photoacoustic imaging (PAI) is an emerging hybrid imaging modality that combines high-contrast optical imaging with high-spatial-resolution ultrasound imaging. PAI can provide a high spatial resolution and significant imaging depth by utilizing the distinctive spectroscopic characteristics of tissue, which gives it a wide variety of applications in biomedicine and preclinical research. In addition, it is non-ionizing and non-invasive, and photoacoustic (PA) signals are generated by a short-pulse laser under thermal expansion. In this study, we describe the basic principles of PAI, recent advances in research in human and animal tissues, and future perspectives.

## 1. Introduction

Modern biomedical imaging has always played an important role in life science research. At present, there is a variety of imaging technologies, including Magnetic Resonance Imaging (MRI), X-ray radiation, Positron Emission Tomography (PET), Single-Photon Emission Computed Tomography (SPECT), ultrasound imaging, and optical microscopy imaging. MRI has a high resolution without radiation hazards and can be performed in multiple directions depending on the diagnostic requirements. However, it is more demanding, and some patients with metallic foreign bodies, for example, cannot undergo this test. Ultrasound is simple and flexible and can be observed in real time. However, due to limited penetration and operator skill, it may not provide effective diagnostic results. PET and SPECT have high sensitivity and specificity for diagnosing tumors and organs. However, they require radioisotopes, which may cause some side effects to the body. In addition, their image resolution is low, which may affect the diagnosis somewhat. X-ray radiography enhances the penetration ability of bone tissue but greatly reduces the resolution ability of soft tissue. X-ray radiography, while enhancing the penetration of bone tissue, significantly reduces the resolution of soft tissues and may be ineffective due to overlapping tissue images, as well as being a radiation hazard. Optical imaging modalities are intuitive and have a high resolution. However, the field of view is often limited in biological examinations, and some treatments that facilitate observation can damage cell activity [1]. As a result, improved methods seek to meet ever-expanding imaging performance requirements, such as resolution, penetration depth, and imaging speed.

Photoacoustic imaging (PAI) is based on the physical phenomenon of the photoa-coustic effect, which is the collection and reconstruction of photoacoustic (PA) signals ex-cited by the illumination light from most tissues. The photoacoustic effect was first pro-posed by Alexander Graham Bell in 1880, who found that acoustic waves could be generated from objects illuminated by modulated sunlight. After discovery, the effect was originally applied by L. B. Kruezer to detect gas constituents until the 1970s [2]. Nowadays, PAI combines the advantages of acoustics and optics, including (i) high optical absorption contrast determined by the optical absorption of different tissues; (ii) excellent spatial resolution and penetration; (iii) real-time imaging; and (iv) only endogenous imaging agents are needed, or paired with exogenous imaging agents, making them promising in biomedical applications [3,4,5].

Depending on the imaging instrument, photoacoustic imaging can be categorized into Photoacoustic Tomography (PAT) and Photoacoustic Endoscopic imaging (PAE), where PAT, in turn, includes Photoacoustic Computed Tomography (PACT) and photoacoustic microscopy (PAM) [6,7]. PAT is the most general method of image reconstruction based on the time-resolved detection of PA signals to visualize the distribution of absorbed optical energy in biological tissue [8]. The advantages of PAT remain the intrinsic optical contrast trait of pure optical imaging and the low-diffraction spatial resolution of pure ultrasound imaging. As for PAM, it accurately recovers the optical absorption distribution of the tissue from the detected photoacoustic signals using a reconstruction algorithm, thus generating clear and accurate images. PA images can be acquired by optically and mechanically scanning a focused ultrasound detector or laser beam. When the focused ultrasound detector is employed, the spatial resolution of PA signals is determined by ultrasound propagation and detection. When a focused laser beam is employed, the spatial resolution is based on the spatial characteristics of laser propagation so that the PAM is defined as Optical Resolution PAM (OR-OAM). As defined in the classification of macroscopic, mesoscopic, and microscopic domains in optical imaging, photoacoustic mesoscopy is regarded as a bridge between photoacoustic macroscopy and photoacoustic microscopy [9]. Photoacoustic mesoscopic detection involves higher ultrasonic frequencies, penetration depths of a few millimeters, and resolutions in the several tens of micrometers.

In this review, we follow a principle-to-application organizational structure, firstly outlining the basic principles and application areas of photoacoustic imaging (PAI) to provide readers with the necessary background knowledge. Then, important existing research results are categorized and sorted out according to the logical thread of classification. We have extensively included important research results in this field in the last decade. This time period was chosen because it covers the key developmental stages of technology and applications in the field of photoacoustic imaging and can fully reflect its latest progress and development trends. In addition, in the process of finding photoacoustic imaging research results, we searched through the Web of Science, which is authoritative and comprehensive. The keywords used include photoacoustic imaging, photoacoustic microscopy, noncontact photoacoustic, and diagnostic imaging. These keywords were selected based on an understanding of the core concepts and hot issues in the field of photoacoustic imaging.

In terms of specifics, we introduce the working principle of PAI and several key elements of PA imaging, including the photoexcitation source and ultrasound transducer. The imaging performance of different imaging modalities in terms of penetration and resolution is discussed, and recent advances in technology development and applications are summarized to provide a comprehensive overview of key information about PAI. Details of PAI are categorized and recapitulated, including the principles and development of ultrasound transducers; molecular PAI using multispectral means; an overview of PAT, PAM, and PA/US; photoexcitation sources; and related applications.

## 2. Principles of Photoacoustic Imaging

### 2.1. Basics of Photoacoustic Effect

**Figure 1:**
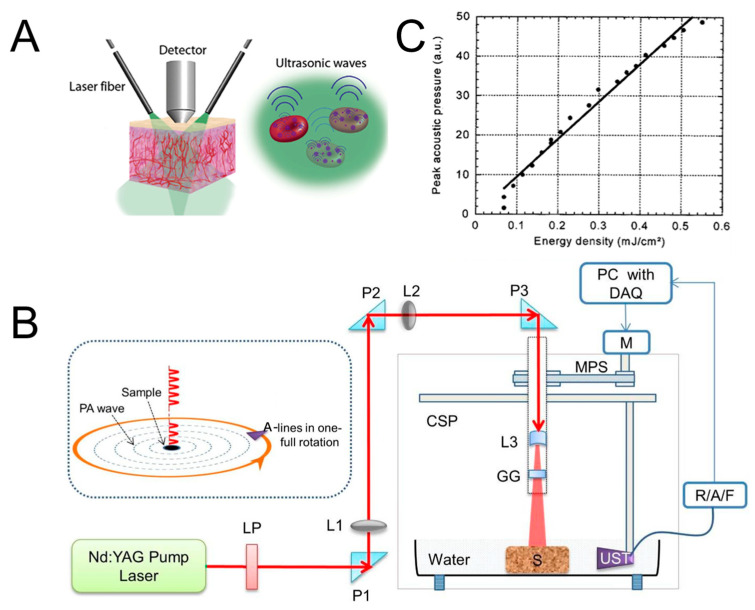
(**A**) Diagram of photoacoustic effect (reproduced from Ref. [10]). (**B**) Schematic diagram of PAI system. LDU: laser driver unit; CSP: circular scanning plate; S: sample; MPS: motor pulley system; M: motor; DAQ: data-acquisition card; R/A/F: ultrasound signal receiver, amplifier, and filter; UST: ultrasound transducer (reproduced from Ref. [11]). (**C**) The dependence of energy intensity per pulse and induced acoustic pressure. Acoustic pressure increases linearly with input energy intensity (reproduced from Ref. [12]).

Light radiation is used to illuminate the tissues of interest and cause the diffuse propagation of photons inside. Diffuse photons are absorbed by specific tissue components, leading to a localized temperature elevation, which triggers thermoelastic expansion. The transient thermoelastic tissue expansion emits pressure waves (ultrasound) whose magnitude is proportional to the deposited light energy, optical absorption coefficient, and thermoelastic properties of the imaged tissue. The initial pressure excited acts as an acoustic source and initiates further acoustic wave propagation in three-dimensional (3D) space. Eventually, ultrasonic waves travel to the surface of the tissue and are detected by nearby transducers to reconstruct the tissue image. The schematic diagram of the photoacoustic effect is shown in Figure 1A. The main components of photoacoustic imaging systems are the light excitation source, ultrasound transducer, sample platform, and data-acquisition and -processing equipment. A complete diagram of the PAI system is shown in Figure 1B. The intensity of the pulse energy and repetition rate can be controlled by the laser driver unit (LDU). The sample and ultrasound transducer (UST) are immersed in water to obtain a lower PA signal attenuation than in air. The laser driver unit can control the intensity of the pulse energy and the repetition rate. The sample can be scanned circularly by the UST driven by a step motor (M). The scanning holder of the UST is installed on a circular scanning plate (CSP) controlled by the motor pulley system. The multifunctional operation of the excitation of the light source, scanning and detection of ultrasonic waves, and processing of PA signals is easily achieved by using a computer. Upgrading all these components will push the development of the PAI system in clinical applications.

The pressure variation in photoacoustic signals is usually limited by thermal and stress constraints within the absorbing tissue. To quantitatively describe the induced initial pressure variations, the assumption that the absorbed optical energy is entirely converted to pressure waves and the volume expansion during the duration of the laser pulse is negligible could achieve the connection between the initially induced pressure $p_{0}\left(r\right)$ and the total absorbed optical energy density $H\left(r\right)$:

(1) $$p_{0}\left(r\right)=\frac{\beta c^{2}}{C_{p}}\cdot H\left(r\right)$$

where r is the location of the sound source, β is the isobaric thermal expansion coefficient, c is the speed of sound in the medium, and $C_{p}$ is the isobaric specific heat capacity. In general, the inhomogeneity of acoustics in different biological tissues is negligible in computing acoustic wave propagation due to the extremely slight difference in sound speed in most soft tissues [1]. As for biomedical application, the absorbed optical energy density could be expressed by the distribution of the absorption coefficient $\mu_{a}\left(r\right)$ that is usually directly related to the concentration of the chromophores:

(2) $$H\left(r\right)=\mu_{a}\left(r\right)\cdot \Phi \left(r,\;\mu_{a},\mu_{s},\;g\right)\;\;$$

where $\Phi \left(r,\;\mu_{a},\mu_{s},\;g\right)$ is the light fluence, $\mu_{s}$ is the scattering coefficient, and g is the anisotropy factor. Considering optical fluence could eliminate the defect of image quality varying with the tissue depth. After the initial pressure distribution duo to the absorbed laser energy is established, the propagation equation of PA waves toward the detected point could be derived for a non-absorbing homogeneous medium as follows:

(3) $$\nabla^{2}p\left(r,t\right)-\frac{1}{c^{2}}\frac{\partial^{2}p\left(r,t\right)}{\partial t^{2}}=-\frac{\Gamma }{c^{2}}\frac{\partial H\left(r,t\right)}{\partial t}$$

where $p\left(r,t\right)$ is the detected pressure waves at the position r in space and time t, and Γ is the PA efficiency of conversion from heat into pressure and generally is regarded as a constant. Thus, the pressure profile $p\left(r,t\right)$ on the detection surface S can be directly related to the initial pressure $p_{0}\left(r\right)$ via a Poisson-type integral:

(4) $$p\left(r,t\right)=\frac{1}{4\pi c}\frac{\partial }{\partial t}\overset{\;}{\underset{S^{\prime }\left(t\right)}{\int }}\frac{p_{0}\left(r^{\prime }\right)}{\left|r-r^{\prime }\right|}dS^{\prime }\left(t\right)$$

where $p_{0}\left(r^{\prime }\right)$ is the spatial distribution of the initial PA pressure at the initial position $r^{\prime }$. $S^{\prime }\left(t\right)$ is the geometric surface center at r with $\left|r-r^{\prime }\right|$ = ct [13]. According to the equation above, the ultrasound detector placed at position r would receive the photoacoustic wave $p\left(r,t\right)$ at time t. More pressure waves could be scanned by the transducer array at these multiple positions r on or close to the boundary of the imaged object, which will acquire more imaging information to enhance the image quality. From the equation above, the PA signals received by each transducer are the integral of the ultrasound waves over the aperture of the detector. When the temporal and spatial measurements are offered simultaneously, a complete reconstruction of a 3D PA source can be achieved [1]. In addition, the dependence of acoustic pressure on energy intensity per pulse also exists. Lihong V. Wang et al. demonstrated the linear relationship between acoustic pressure and input energy density through the detection of ultrasound waves for gel buried in a mineral oil bath, as shown in Figure 1C [12]. 

### 2.2. Imaging Instrumentation

#### 2.2.1. Light Excitation Source

In PA imaging, the most suited wavelength range is between 532 nm and 1064 nm, which is part of the visible and near-infrared (NIR) spectrum. Compared with the visible spectrum (VIS), NIR illumination can achieve a greater tissue penetration depth due to its weaker optical attenuation and light scattering to maintain an excellent lateral resolution. Moreover, the American National Standards Institute (ANSI) permits a stronger NIR light intensity on the tissue surface [14]. For the light excitation source, usually a Nd:YAG pump laser either pumps a dye laser or an Optical Parametric Oscillator (OPO) laser to generate light in the NIR wavelengths or visible light. However, these types of lasers are bulky (often requiring an optical table to house the laser), expensive, and slow (the typical pulse repetition rate for such lasers is 10–20 Hz with ~100 mJ of energy per pulse), making the imaging system difficult to translate into clinical applications. Therefore, new excitation light sources are constantly being developed by different research teams to meet the requirements of compactness, affordability, and higher frame rates. Paul et al. presented an OA system based on a miniaturized pulsed laser diode, which could offer NIR pulses at ~803 nm. Kathyayini et al. [15] reported up to 7000 fps PAT imaging using a low-cost, lightweight pulsed laser diode (PLD). The PLD has a maximum pulse repetition frequency of 7000 Hz and a maximum pulse energy of ~1.4 mJ. The capability of high-frame-rate imaging is achieved by imaging the black ink flow at various flow rates. 

**Figure 2:**
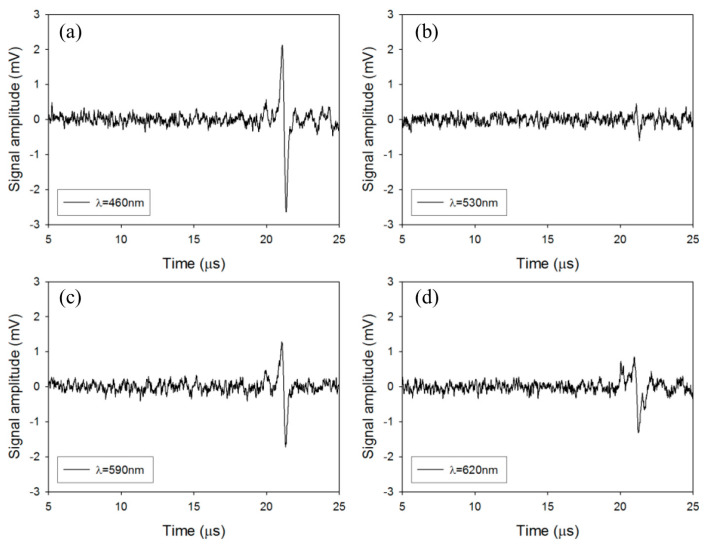
Photoacoustic signals generated for PA amplitude image excited with a 460 nm LED (**a**), 530 nm LED (**b**), 590 nm LED (**c**), and 620 nm LED (**d**) (reproduced from Ref. [17]).

In the visible light range of 400 nm to 632 nm, T. J. Allen proposed high-power light-emitting diodes (LEDs) as an excitation source for biomedical applications. The high-power LEDs could drive all four wavelengths at once and acquire multiwavelength data sets simultaneously. While specific components of biological tissues could be detected in single wavelength images, the volumetric imaging of photoabsorbing agents typically requires differentiation of these agents on top of spectrally varying background absorption. The multiple spectrum principle is where tissues are irradiated with pulses of different wavelengths in a time-sharing manner at certain durations, and the wavelengths are selected to sample a spectral characteristic in the absorption spectrum [16]. Thomas J. et al. [17] explored a new alternative to conventional Q-switching using visible LEDs. In common with laser diodes, LEDs provide the advantages of compact size, low cost, and high efficiency. Photoacoustic signals were generated for a PA amplitude image excited with a 460 nm LED (Figure 2a), 530 nm LED (Figure 2b), 590 nm LED (Figure 2c), and 620 nm LED (Figure 2d). However, laser diodes suitable for pulsed photoacoustic generation are typically available only at wavelengths greater than 750 nm. By contrast, LEDs are readily available at visible wavelengths below 650 nm where hemoglobin absorption is significantly higher through superficial vascular imaging in physiologically realistic vascular phantoms. The study demonstrates that LEDs could find application as inexpensive and compact multiwavelength excitation sources for imaging superficial vascular anatomy.

With the continuous development of imaging technology, multispectral imaging based on polarization-holding optical fibers has shown great potential for application in many fields due to its unique advantages. Wang et al. propose using the “depolarization” phenomenon of multispectral polarized light in living tissues to achieve video optical detection of human skin. This method requires only a few lines of MATLAB code and is highly reproducible and easy to use [18]. Wang et al. also realized the coupling and docking of a superluminescent light-emitting diode and the online fabrication of a fiber polarizer on a 40 μm bias-preserving fiber. They constructed a simple polarized interferometer device, which can measure the beat length and the birefringent temperature coefficient simultaneously using modulation spectra, which are elaborated and verified from theoretical and experimental perspectives [19]. Wang et al. introduced a temperature self-compensating multi-parameter sensor based on a bias-preserving fiber Bragg grating, which can simultaneously measure multiple parameters, such as the temperature, strain, and refractive index, and has the advantages of high sensitivity, a fast response speed, and good stability [20]. Tang et al. developed a polarization-sensitive optical coherence tomography technique based on a polarization-holding optical fiber capable of high-resolution, three-dimensional imaging of biological tissues with significant advantages, such as being non-invasive and label-free [21]. Zhang et al. proposed a high-sensitivity multispectral imaging technique based on a polarization-holding optical fiber capable of high-resolution, multispectral imaging of tiny objects. This technique has the advantages of high sensitivity and fast response speed [22]. Multispectral imaging using polarization-holding optical fibers has achieved significant results. However, there is still room for further exploration regarding technology optimization, performance enhancement, and application expansion. In the future, it is expected to play a more critical role in more complex scenarios and finer demands, generating more innovations and breakthroughs in related fields.

#### 2.2.2. Ultrasound Transducer

**Figure 3:**
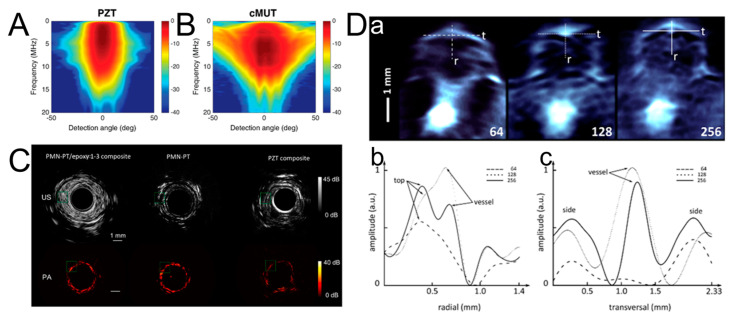
The spectral sensitivity of PZT (**A**) and cMUT (**B**) as a function of the angle (reproduced from Ref. [31]). (**C**) US and PA images of rat rectum with PMN-PT/epoxy 1-3 composite, PMN-PT, and PZT composite (reproduced from Ref. [33]). (**D**) (**a**) The reconstructed PA image with 64-, 128-, and 256-element transducer. (**b**) PA amplitude diagram of vessel along the radial direction. (**c**) PA amplitude diagram of vessel along the transversal direction (reproduced from Ref. [41]).

An ultrasonic transducer is an important component of PA signal detection. Most demonstrations of PAI currently use commercial conventional US transducers based on piezoelectrics, such as a Piezoelectric Transducer (PZT) or PZT-based composites [23], which have superiority in terms of their comparatively low cost of implementation and highly sensitive measurements [24]. In addition to PZT-based composites, capacitive micromachined ultrasound transducers (cMUTs) can also generate and direct ultrasonic waves [25]. cMUTs have recently been disclosed and recognized as a promising transducer technology since their intrinsic behavior makes them a complementary solution to conventional PZT in certain applications. The feasibility of using cMUTs for PAI has been demonstrated by several research groups [26,27,28]. Moreover, cMUTs achieve more favorable results regarding imaging quality, including contrast and the Signal-to-Noise Ratio (SNR) [29,30]. cMUTs, however, operate at a higher voltage and may result in a noisier image due to the smaller capacitance. However, their bandwidth can be up to twice that of piezoelectric detector arrays, so they can be used as a complementary solution for some miniaturized ultrasound devices. Furthermore, Johannes Rebling et al. developed a calibrated optoacoustic source to characterize the frequency dependence of the angular response for conventional PZTs and cMUTs [31]. Figure 3A,B shows the angular sensitivity performance of PZTs and cMUTs. cMUTs have a large detection angle and frequency range, proving their ultra-sensitivity in the 2 to 6 MHz frequency range. A study by Johannes Rebling et al. showed that cMUTs also have outstanding advantages in improving the imaging quality of PAI systems [32]. Yan Li et al. developed a miniature single-element 32 MHz Lead Magnesium Niobate–Lead Titanate (PMN-PT) epoxy 1-3 composite-based ultrasonic transducer. Comparing some PMN-PT and lead zirconate composite ultrasonic transducers, this ultrasonic transducer exhibits a higher SNR and bandwidth [33]. The imaging probes of the transducer were fabricated by utilizing three materials to detect the US and PA signals to image rats. As shown in Figure 3C, the imaging probe qualities of PMN-PT and PZT, as well as PMN-PT/epoxy 1-3 composites, are demonstrated, proving the applicability of the cMUT to ultrasound transducers. Focused ultrasound transducers and ultra-wideband ultrasound transducers are also covered and have been the subject of numerous studies. The design and performance of focused ultrasound transducers have been analyzed in detail by Keller et al. This study addressed the optimization of the transducer geometry, the influence of material properties, and the resulting imaging quality and therapeutic effects [34,35]. Bian et al. reviewed the material-selection and fabrication processes used to make ultra-wideband ultrasonic transducers and evaluated their frequency response and bandwidth characteristics [36]. Lee et al. explored innovative transducer configurations, thereby enhancing the sensitivity and resolution of ultrasonic imaging, leading to potential advances in medical diagnostics and non-destructive testing [37,38]. Odabaee et al. used advanced modeling and simulation techniques to understand and predict the behavior of ultrasonic transducers, which led to the design and optimization of transducer systems [39]. Hübschen et al. explored the practical applications of ultra-wideband ultrasonic transducers for characterizing material properties and detecting defects, emphasizing their importance in industry and research [40]. These studies have significantly advanced the understanding and development of ultrasonic transducers, opening up new possibilities to enhance performance and expand applications in different fields. Since the transducer performance governs the image quality, the transducer specification should be customized to optimize the performance in PAI. 

Furthermore, the geometry of the transducer array and the number of transducers also affect the imaging performance. Linear-array-based PAI systems allow images to be acquired with just a few laser pulses and provide significantly higher frame rates. For a scanned system of a single transducer, long acquisition times are needed, which is a significant challenge in obtaining high-quality images under the control of animal physiological parameters, motion, and anesthesia. The improved imaging performance obtained by using more sensors was studied by Alexander Dima et al. Using 64-, 128-, and 256-element transducers to image the same phantoms and animals, the results demonstrated that the increasing number of detectors could improve the resolution and overall image quality and showed that the image quality of the 256-detector array was significantly improved [41]. Figure 3D-a provides the PA image obtained by the 64-, 128-, and 256-element systems on the spinal disk. The amplitude image obtained from the radial and transversal direction of the image profiles in Figure 3D-a is shown in Figure 3D-b,D-c, demonstrating that the adjacent vessels could be resolved from the edge in both the radial and transversal directions using a 256-element transducer. The vessel in the transversal direction could be distinguished with 128- and 256-element transducers but not with 64-element transducers. The vessel and adjacent top edge in the radial direction fuse when 64-element and 128-element transducers are utilized. The clear edge of the adjacent vessel in the radial direction could only be distinguished with a 256-element transducer. In addition, the field of view could be enhanced without sacrificing sensitivity when increasing numbers of detectors were utilized. 

As mentioned earlier, the geometry of the transducer arrays affects the imaging performance; therefore, transducer arrays with different geometrical arrangements have been explored to improve the imaging speed. Moreover, these array setups need to satisfy the prerequisites of sensitivity and bandwidth-frequency characteristics of PAI [42]. Roger J. Zemp et al. first proposed a 30 MHz ultrasound array system for imaging small blood vessels in rats, achieving a lateral resolution of 100 μm, an axial resolution of 25 μm, and an imaging depth of 3 mm [43]. Comparing spherical and planar probe geometries, planar geometries are more widely used. Hui Wang et al. developed an ultrasound Fresnel Zone Plate (FZP)-transducer-based PA imaging system for biological tissue imaging [44]. The FZP transducer can be scanned in a straight line or in a planar plane, which is more flexible than traditional circular scanning modes, and thus it can be applied to different parts of the human body for the live imaging of absorptive structures such as blood vessels or tumors [35]. While meeting the prerequisite of adapting the sensitivity and bandwidth-frequency characteristics of the PAI, these two transducer array shapes enable increased imaging speeds by improving the breadth of applications and the flexibility of scanning modes. 

### 2.3. Imaging Performance

#### 2.3.1. Imaging Algorithms

In order to recover the optical absorption properties of tissues, many algorithms have been developed to reconstruct images accurately or approximately using full-view data [45]. Based on data-processing methods as well as performance characteristics, we classify commonly used imaging algorithms into three categories: back-projection algorithms, compressed perception algorithms, and iterative reconstruction algorithms.

The traditional Filtered Back-Projection (FBP) algorithm requires a large number of data acquisitions and measurements, resulting in long scanning times. In addition, it cannot achieve full coverage of the entire tissue surface. The back-projection algorithm is widely used as a computationally simple and faster image reconstruction algorithm for linear, planar, curved, circular, and hemispherical sensor arrays. In most cases, the back-projection algorithm is both fast and reliable [46]. However, since it is not modality-based, it may introduce artifacts in the reconstructed image in case of incomplete or noisy projection data [47]. In this case, a compressed perception algorithm or an iterative reconstruction algorithm can be used to eliminate the artifacts. Among them, iterative reconstruction algorithms can compensate for the sensitivity of inverse-projection algorithms to noise and incomplete data [48,49]. However, iterative reconstruction algorithms are more complex and require more computational resources and time. To address these limitations, imaging algorithms based on compressed sensing (CS) theory can achieve artifact-free imaging with finite view acquisition [50], or in the case of sparse or compressible images, the number of measurements is much lower than that required by mountainous non-sampling theory [51]. This approach allows the sparse signal to be recovered from unsampled measurements. In addition to the three image reconstruction algorithms described above, there are various other excellent imaging algorithms. For example, the time-reversal (TR) algorithm is very effective for closed surfaces that are completely open around the object under test in specific cases [52,53,54]. Therefore, we should synthesize the actual situation when designing image reconstruction algorithms and reasonably select and match the imaging algorithms so as to achieve the best effectiveness.

In conclusion, various PA reconstruction algorithms have been studied in order to optimize the image information. In medical imaging, there are also situations where multiple algorithms work together [55]. For example, in emergency situations, an inverse-projection algorithm can be used to quickly acquire a preliminary image, and then, an iterative reconstruction algorithm can be used for more accurate diagnosis [56,57]. In some high-end imaging devices, compressed perception algorithms can be used together with iterative reconstruction algorithms to obtain high-quality images while reducing the radiation dose to the patient [58,59].

#### 2.3.2. Contrast Agents

**Figure 4:**
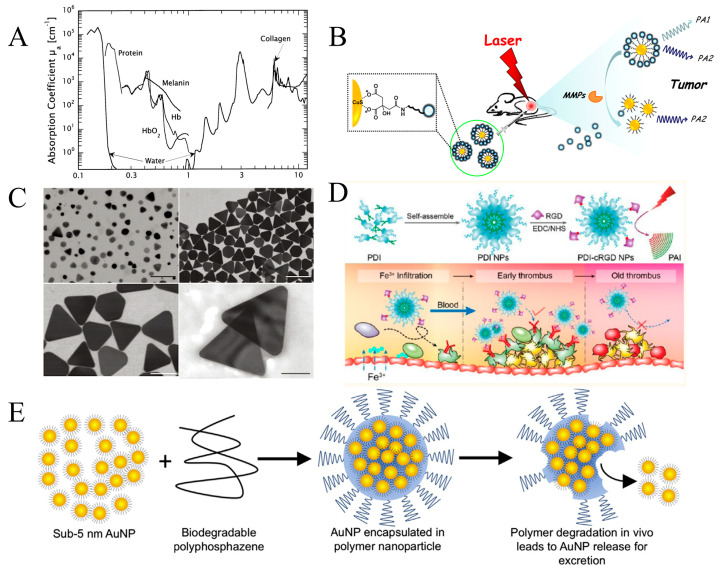
(**A**) Optical absorption coefficient of principal tissue chromophores (reproduced from Ref. [91]). (**B**) The schematic illustration of the CPQ nano-probe activated by MMPs. The conjugated Black Hole Quencher 3 (BHQ3)–peptide–CuS could be cleaved after exposure to MMPs so that the BHQ3 could be released from nanoparticles (reproduced from Ref. [79]). (**C**) Schematic diagram of synthesized silver nanoplates with rounded and more stable tips (reproduced from Ref. [82]). (**D**) Schematic illustration of the preparation of cRGD-PDI NPs and specifical mechanism for lighting early thrombus (reproduced from Ref. [83]). (**E**) Schematic depiction of biodegradable gold nanoparticles. The contrast agent is generated by small AuNPs incorporated into a biodegradable PCPP to achieve diagnostic potential as well as be degraded in vivo into harmless byproducts for excretion after some period (reproduced from Ref. [84]).

Photoacoustic imaging can be performed using a variety of endogenous and exogenous contrast agents. The endogenous chromophores with different absorption spectra inside body tissue could be utilized, including melanin, water, fat, hemoglobin, proteins, and DNA, as shown in Figure 4A, which dominates the light absorption with varying wavelength ranges for different tissues. The structural and functional information of the microvasculature could be obtained due to the rich content of hemoglobin [60,61]. Strong light absorption is presented by melanin in the skin, retina, and melanoma cells over a broad range of wavelengths [62,63]. In general, the diseased tissue causes corresponding changes in tissue constituents. PAI has the potential to visualize and quantify these lesion tissues when they are compared with normal surrounding tissues. PAI based on endogenous contrast has been used in various tissue imaging, such as atherosclerosis, breast cancer, prostate cancer, and human skin [64,65,66,67]. For some tissue components with similar light absorption coefficients, PAI presents a weak imaging contrast or the inherent light absorption agent in some specific tissues. Therefore, exogenous imaging agents are required to enhance the contrast and facilitate deeper tissue imaging [68,69,70]. Stable and efficient contrast agents play a significant role in PAI applications, which can be delivered to or retained at the target site, and no photobleaching occurs after prolonged laser exposure [71]. Exogenous contrast agents utilized for PA imaging generally consist of highly absorbent dyes or nanoparticles that can be conjugated to a targeting moiety of interest [72,73,74].

Targeted imaging agents can generate specific information on molecular or cellular processes. Nontargeted agents usually exist due to a leaky vasculature and enhanced permeability in tumor tissue. Many imaging agents can be used to improve the specificity and contrast of biological tissues. The choice of a particular imaging agent depends on the biological tissue being imaged. Various biological characteristics of imaging agents should also be considered, such as the size, shape, toxicity, stability, surface chemistry, and molecular target. Imaging agents used for biological tissue imaging include Indocyanine Green (ICG), Evans’s blue, and methylene [75,76,77,78]. Kai Yang et al. introduced a CuS–peptide–BHQ3 (CPQ) nano-probe, which shows a strong photoacoustic signal at the wavelengths of 680 nm and 930 nm [79]. The CuS nanoparticles and Black Hole Quencher 3 (BHQ3) molecules are conjugated together through a cancer-related Matrix Metalloproteinase (MMP)-cleavable peptide linker for the in vivo detection of specific enzyme activity. Compared with conventional MMP detection, PAI based on the CPQ nano-probe could significantly enhance tissue penetration and spatial resolution during imaging owing to its unique mechanism [80,81]. The working mechanism of the CPQ nano-probe is shown in Figure 4B. The CPQ nano-probe could be recognized by the protease releasing free BHQ3 and leaving bare CuS nanoparticles, which would remain inside the tumor for a longer period. Synthesized silver nanoplates have been reported as a molecularly sensitive photoacoustic contrast agent for in vivo application, as shown in Figure 4C [82]. The benefits of biocompatibility and -stability and antibody conjugation satisfied the requirement of imaging performance. 

The accurate diagnosis of early thrombus is greatly limited due to the absence of an endogenous PA signal of hemoglobin. Organic semiconducting nanoparticles (NPs), which are self-assembled by amphiphilic Perylene 3,4,9,10-tetracarboxylic Diimide (PDI) molecules and are modified with Cyclic Arg-Gly-Asp (cRGD) peptides, are exhibited as a PA contrast agent (cRGD-PDI NPs) in the diagnosis of early thrombus by Cao Cui et al. [83]. The cRGD-PDI NPs proved to be a good biomarker in terms of the depiction of the profile, size, and conformation of early thrombus as well as the real-time monitoring of the obstructive degree of the thrombus in blood vessels and the thrombolysis effect. Figure 4D-a shows the preparation process of cRGD-PDI NPs, which includes mainly the assembling of the PDI molecule and modification of PDI NPs with cRGD. The mechanism for specifically lightening early thrombus by PAI is shown in Figure 4D-b. Gold nanoparticles (AuNPs) encapsulated into biodegradable Polydi (Carboxylatophenoxy) Phosphazene (PCPP) with biocompatibility and biodegradability are developed as imaging contrast agents by Rabee Cheheltani et al. [84]. Compared to biodegradable gold nanoparticle platforms [85,86,87], the advantages of Au-PCPP are the tunability of particles sizes and high gold-to-polymer ratios. The encapsulation and degradation route of Au nanoparticles is shown in Figure 4E. Small AuNPs are gathered into a biodegradable PCPP as contrast agents. The resulting Au-PCPP would be degraded into smaller AuNPs for excretion. Palladium (Pd) Nanosheets (PNSs) were reported to have high photothermal efficiency and catalytic properties [88,89]. Liming Nie et al. [90] presented the application for optical diagnostic imaging utilizing PNSs with high photothermal stability and an ultrathin structure, which demonstrated the feasibility of Palladium Nanosheets (PNSs) as a novel class of probes for in vivo tumor perfusion imaging.

#### 2.3.3. Imaging Resolution

The spatial resolution is one of the most important parameters used to evaluate imaging performance. Regardless of PAT, PAM, and PAE, the optimal resolution is ultimately limited by ultrasonic diffraction. The penetration depth is generally limited by optical and acoustic attenuation. Compared with acoustic attenuation, optical attenuation is dominant [6]. For optical attenuation, photon propagation in soft tissue has a dissipation limit (~10 cm in tissue) from the quasi-ballistic regime (typically ≤1 mm in tissue) to the diffusive regime (typically ≥10 mm in tissue) [80]. Depending on the wavelength, the light penetrates to a certain depth. Multiple scattering and absorption in the tissue occurs. The acoustic pressure is generated by the specific chromophores. For acoustic attenuation, a higher ultrasound frequency can achieve a better resolution but a smaller imaging depth. When the imaging depth is reduced, its spatial resolution is improved in an approximate proportion. S.A. Telenkov et al. presented a methodology for the depth-selective imaging of tissue chromophores [92]. They introduced chirped optical excitation to provide a linear frequency-modulated response to clearly and accurately measure the depth of tissue chromophores, and the direct relationship between the chromophore depth and spectrum of PA signals was established to achieve a depth-selective tissue image. Additionally, the use of exogenous contrast agents can enhance the signal strength and improve the imaging depth [93]. In general, the ultrasound signals undergo frequency-dependent attenuation in tissue, about 0.5 dB/cm/MHz, on its propagation toward the transducer detectors [94]. The temporal pulse width of ultrasound signals is proportional to the chromophore diameter [95], in which the small size of the absorber generates short pulses with a high frequency. Due to frequency-dependent acoustic attenuation, the high-frequency PA signal undergoes a greater attenuation in tissue [96,97], which affects the spatial resolution and image depth.

Moreover, the duration of the laser pulse also affects the temporal pulse width. In ideal conditions, in order to meet the confined stress condition, the tissue heating time is supposed to be short enough since acoustic pressure reaches its maximum in a relatively short time. Besides acoustic attenuation, the bandwidth of the transducer also limits the frequency range of PA signals that are received. 

The spatial resolution depends ultimately on the frequency content of the acoustic wave arriving at the detector. However, the bandwidth of propagating PA signals limits the maximum frequency content of the PA signals, thus defining the ultimate spatial resolution limit that can be achieved [6]. A common practice is to match the center frequency of the transducer to the expected PA frequency, and a greater bandwidth should be chosen. The bandwidth of the generated pressure wave can be predicted from the duration of the excitation pulse and the geometrical properties of absorbers in tissue. The narrower pulses can excite broader signals. After determining the imaging depth according to the requirements, the spatial resolution factor should be carefully considered. For PAT, the spatial resolution can be adjusted with optical and acoustic configurations. Shorter excitation wavelengths and a tighter optical focus provide a good lateral resolution of OR-PAM. A wider transducer bandwidth provides a better axial resolution of OR-PAM and AR-PAM [98,99]. A higher central ultrasonic detection frequency is beneficial to the axial resolution of AR-PAM. An increase in spatial resolution is based on the loss of penetration depth in all cases. The weaker optical scattering of NIR light can provide a better focusing performance compared with VIS light, which results in finer lateral resolution in NIR-OR-PAM over VIS-OR-PAM [14]. Due to the differences in imaging modality, the magnitude of the spatial resolution and imaging depth are usually very different. Application practices for the imaging resolution and depth of penetration are summarized in Table 1 below.

## 3. Application

In the previous section, we categorized the principles of photoacoustic imaging, photoacoustic imaging components, and their characteristics. The advantages of photoacoustic imaging over other imaging systems and research advances have been summarized at the theoretical and technical levels. Therefore, next, we demonstrate the advantages of photoacoustic imaging systems in the context of the research progress and the results of their application to the characteristic diseases involved in common imaging examinations in the clinic, covering several departments (skin, joints, breast tumors, vascular diseases, cervical cancer, animal organs, and brain imaging). These specific areas of human application are important in clinical practice, and the use and development of imaging technology to examine them is significant in enhancing the diagnostic and therapeutic outcomes for patients. Through the in-depth investigation and review of these applications, we intend to provide valuable references and insights for research and clinical practice in related fields.

### 3.1. Dermatologic Imaging and Joint Imaging

**Figure 5:**
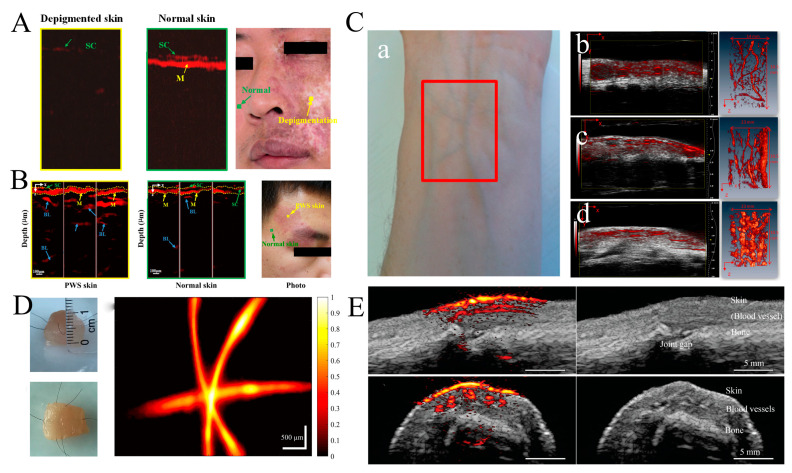
(**A**) PA images and photographs of skin with pigmentation and depigmentation in epidermal structures (reproduced from Ref. [106]). (**B**) PA images and photographs of PWS skin and normal skin (reproduced from Ref. [106]). (**C**) In vivo PA/US images of the human forearm obtained with 40, 21, and 15 MHz frequency transducer probes at 1064 nm wavelength. (**a**) Photograph of forearm skin from the subject. Fused PA/US imaging acquired with 40 (**b**), 21 (**c**), and 15 (**d**) MHz frequency transducer probes, as well as corresponding (Maximum Intensity Projection) MIP images through the PA volumes of the human forearm (reproduced from Ref. [107]). (**D**) Schematic illustration and PAM image of chicken breast piece with crossing hairs inserted ~1 mm below the surface. The resolution of hairs is ~15 dB SNR (reproduced from Ref. [108]). (**E**) Photoacoustic/ultrasound images of a human proximal interphalangeal joint in sagittal and transverse planes. On the right side, the anatomical structures are indicated by ultrasound imaging (reproduced from Ref. [112]).

In order to avoid over-treatment and under-treatment in clinical skin treatment [100,101], it is necessary for skin disease to be evaluated and analyzed, which requires skin structure information and lesion parameters. Traditional skin-imaging techniques always have some drawbacks, including a low resolution, limitations in biological light scattering, and low contrast [102,103,104]. Due to its advantages in imaging depth and resolution, the study of skin imaging utilizing PAI has attracted great attention [105]. Xu Dong developed Photoacoustic Dermoscopy (PAD) for the high-resolution imaging of human skin [106]. The technology of PAD achieved an axial resolution of 31.5 μm and a lateral resolution of 3.98 μm. The epidermal structures of human skin are revealed clearly due to the different optical absorption characteristics, including the stratum corneum (SC) and melanin layers (M). In addition, pigmentation and depigmentation in human skin are investigated. Figure 5A shows PA images of pigmented skin and the surrounding normal skin from a facial pigmentation patient, as well as the examined locations. Compared to normal skin, the depigmented area of the patient’s face almost could not be used to find PA signals of epidermis and melanin. PA dermoscopy has also been utilized to image the lesioned skin of one scarlet-lesion-type patient. The PA signal mapping of Port Wine Stain (PWS) and normal skin, as well as the detection locations, are shown in Figure 5B. The dotted line represents the surface tissue of the skin containing stratum corneum and pigment layers. Blood vessels are more easily imaged via PA detection due to the presence of hemoglobin. Compared to blood vessels in normal skin, the blood vessels in the affected skin show an irregular arrangement. The diameter and depth of the blood vessels within PWS lesion skin are much larger than those found in normal skin. After obtaining information on the diseased vessels, an appropriate treatment scheme can be selected to improve the cure rate of PWS. 

Haroon Zafar et al. proposed a high-frequency multielement linear-array transducer combined with a multichannel collecting system to image the volumetric structure of human subcutaneous blood vessels [107]. The vivo images of a human forearm were created under 800 nm and 1064 nm wavelengths with 40, 21, and 15 MHz frequency transducer probes. Figure 5C exhibits the fused PA/US single vertical (x-y) B-scans of forearm skin obtained at a 1064 nm wavelength using 40, 21, and 15 MHz frequency transducer probes. The gray scale of the US image indicates the layered skin morphology. The red PA signals show the distribution of blood vessels throughout the dermis and underlying subcutaneous tissue. The Maximum Intensity Projection (MIP) of PA volumes is shown on the right of Figure 5C using 40, 21, and 15 MHz frequency transducer probes. As the frequency declines, so does the resolution of the image, and some of the microvascular structures are masked at low frequencies. Compared to imaging at a 800 nm wavelength, imaging at 1064 nm achieves higher penetration depths due to the lower optical attenuation in blood. It can be predicted that linear-array-based microcirculation PA imaging will play an important role in the diagnosis and treatment of various skin and subcutaneous tissue diseases.

Mohsen Erfanzadeh et al. developed a laser scanning laser-diode-based photoacoustic microscopy system, which meets the requirement of rapid image acquisition in clinical vivo PAM applications [108]. The system achieved fast imaging of 370 A-line per second compared to other laser-diode-based PAM systems [109,110,111]. The lateral resolution of the system is estimated to be 21 μm when using the edge-diffusion function. The axial resolution is 339 μm. Figure 5D shows an image of crossed black human hair inserted 1 mm below the surface of a chicken breast. The left side of Figure 5D shows the side and overhead views of the chicken breast piece. The right side of the figure shows the PAM image of crossing hairs inserted ~1 mm below the chicken breast surface. The color bar represents the normalized PA amplitude. K. Daoudi et al. proposed a handheld probe of dual-modality ultrasound/photoacoustic imaging, which assesses the imaging performance of the human finger joint [112]. The photoacoustic and ultrasound imaging modes are used successively to reconstruct images of the sagittal and transverse plane of the index finger’s proximal interphalangeal (PIP) joint, as shown in Figure 5E. The grayscale pixels are reconstructed using ultrasound data, whereas the red pixels are reconstructed using photoacoustic data. Skin and the underlying bone and joint gap are shown in the sagittal ultrasound image. Combined PA/UA images show the skin and subcutaneous blood vessels. The penetration depth is up to 15 mm for 0.5 Hz frame rate imaging when studying more complex models. The compact and handheld hybrid photoacoustic and ultrasound system greatly reduces the cost of clinical applications, which lays the foundation for future US-PA imaging systems [113].

### 3.2. Breast Tumor Imaging and Vascular Disease Detection

Breast cancer has always been a great threat to women’s health, and mammographic detection is regularly performed to help find potential malignancies in their early stages, which can greatly improve the cure rate of the disease. Due to the high contrast and high spatial resolution of PA imaging, the vascular structures that lie within and surround a tumor can be displayed distinctly based on the blood-oxygen level to differentiate malignant from benign masses. Jason Zalev et al. reported the potential benefits and utility of the clinical PA imaging of breasts using the Imagio Breast System [114,115]. The system could perform two-dimensional functional photoacoustic imaging of tissue and vascular structure in real time, as well as ultrasonic grayscale images representing tissue anatomy and morphology. Typically, the density of blood vessels around malignant tumors is higher than that around benign tumors, and the binding rate of blood oxygen is also higher. With the advantages of PAI, the presence or absence of specific features of malignant tumors can be detected to help distinguish benign from malignant tumors.

**Figure 6:**
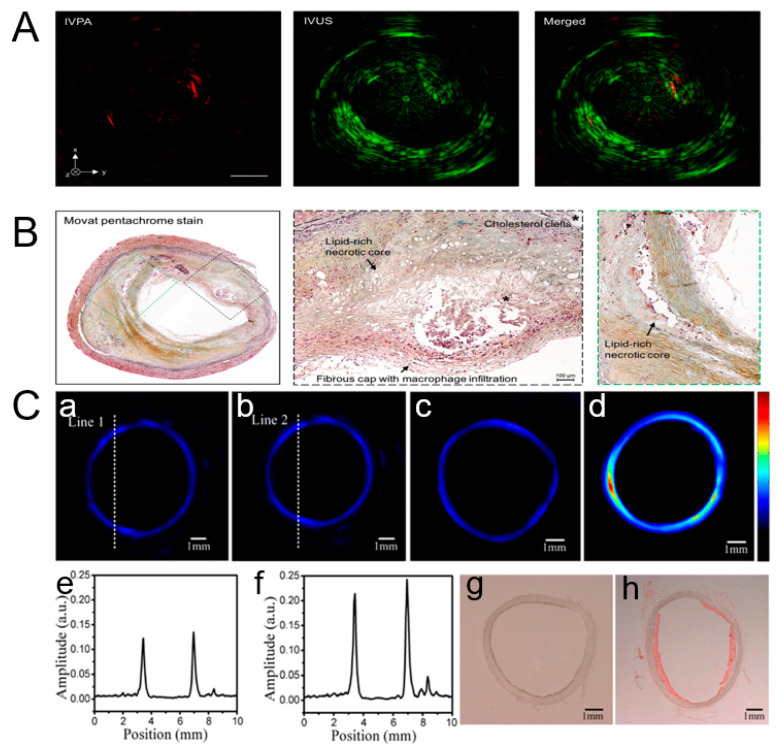
(**A**) Cross-sectional images of IVPA and IVUS and merged images of IVPA-US (reproduced from Ref. [116]). (**B**) Histopathology stained with special dye and magnified images of lipid deposition site (reproduced from Ref. [116]). (**C**) PA images of a normal vessel by utilizing single-element (**a**) and dual-element (**b**) transducer as well as distribution of PA amplitude along the dashed line in (**a**,**b**). PA images of a normal vessel (**c**) and an atherosclerotic plaque (**d**) as well as corresponding bright-field optical images of a normal vessel and an atherosclerotic plaque. (**e**–**h**) Lipid-rich plaques in the atherosclerotic vessel sample stained with oil red (reproduced from Ref. [117]).

Jie Hui et al. reported a portable intravascular photoacoustic–ultrasound (IVPA-US) system, which achieved 25 frames per second in a real-time display mode [116]. Furthermore, the system demonstrated ex vivo imaging capability for an atherosclerotic human coronary artery at 16 frames per second. Figure 6A shows two sites of lipid deposition at the 2 and 8 o’clock positions in the IVPA channel due to the stronger optical absorption coefficient of intravascular lipids than other tissue components. Intravascular Ultrasound (IVUS) can show the cross-sectional view of artery morphology. The merged image shows the position of the lipid-rich core in the artery morphology. Figure 6B offers an overall image of histology and an enlarged view of the lipid deposition site in the corresponding dotted box. The two lipid-rich necrotic cores are apparent, as identified by the loss of matrix and cholesterol clefts. Integrating radiographic and histological features, this plaque can be identified as advanced fibroatheroma. The image result confirms that the motion artifacts induced by cardiac pulsation do not occur at a 16-frames-per-second imaging speed. Simultaneously, the image quality is not compromised while maintaining this speed. Ji et al. developed an intravascular confocal PA endoscope with symmetrically aligned dual-element ultrasonic transducers to image an atherosclerotic vessel [117]. Unlike the IVPA-US system, the intravascular confocal PA endoscope achieved an improved resolution and SNR. The impact of optical scattering on the resolution is reduced by combining focused laser excitation and focused acoustic collection. Figure 6C-a,C-b indicate imaging of a normal vessel in the New Zealand White (NZW) rabbit model by utilizing single-element and dual-element transducers, respectively. Figure 6C-e,C-f show the corresponding PA signal amplitude of normal vessels along the dashed line. Compared with a single-element transducer, the enhanced PA signal amplitude can be observed by the dual-element transducer. PA images of normal and atherosclerotic vessels in the NZW rabbit model are shown in Figure 6C-c,C-d. Figure 6C-d shows the amplitude distribution of PA signals based on the difference in the optical absorption of atherosclerotic vessel components. The lipid-rich regions with higher optical absorption coefficients generated higher PA signals. Figure 6C-h shows lipid-rich plaques in the atherosclerotic vessel sample stained with oil red. However, no lipid is found in the normal vessel, as shown in Figure 6C-g. The intravascular confocal PA endoscope with a symmetrically aligned dual-element transducer has a higher sensitivity and spatial resolution, which shows huge potential in detecting vascular disease.

### 3.3. Cervical Cancer

**Figure 7:**
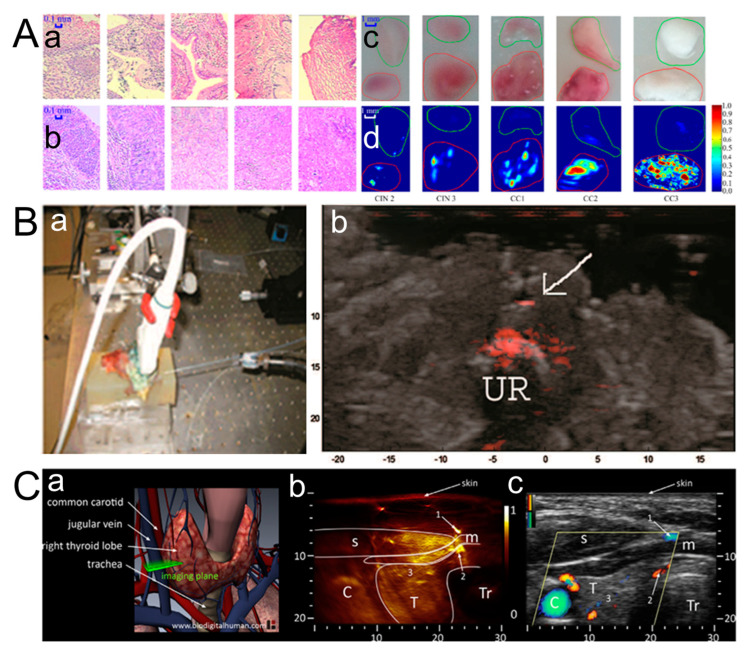
(**A**) (**a**,**b**) Histological results for the normal cervical tissue and tissue lesion, (**c**) tissue image corresponding to the DMAP images, and (**d**) Depth Maximum Amplitude Projection (DMAP) image (reproduced from Ref. [118]). (**B**) (**a**)A clear anatomical view is produced by combining photoacoustic-based cell-specific targeting with ultrasound imaging, (**b**) Imaging setup including the ultrasound probe and fiber illumination inserted through the urethra (reproduced from Ref. [67]). (**C**) (**a**) anatomy of thyroid gland including cardiovascular and respiratory system. (**b**) PA image of the left thyroid lobe of volunteer. The vascular features of skin [71,124], muscles, and within the thyroid lobe [125] are shown through leveling and normalizing from 0 to 1. (**c**) Ultrasound cross-sections of the left thyroid lobe. The superimposed areas in color represent directional power Doppler signals. C: carotid; T: thyroid; Tr: trachea; s: sternocleidomastoid muscle; m: infrahyoid muscle (reproduced from Ref. [122]).

The diagnosis of cervical cancer (CC) tends to require a biopsy, which increases costs in terms of both time and money. The photoacoustic diagnosis of CC proposed by Kuan Peng et al. achieved intact scanning both around the external orifice and in the cervical canal instead of only detecting superficial lesions around the external cervical orifice [118]. Histological results for the normal cervical tissue and tissue lesion are shown in Figure 7A-a,A-b. A Depth Maximum Amplitude Projection (DMAP) image is shown in Figure 7A-d, which can roughly represent the optical absorption distribution of tissue to help evaluate the angiogenesis level for diagnosing CC. Figure 7A-c shows the tissue image corresponding to the DMAP images. The margins of cervical lesions and normal tissue are drawn manually with red and green lines, respectively, in the photo and DMAP images. The development of cervical cancer leads to the abnormal generation of new blood vessels [119,120]. Compared to normal tissue, the optical absorption of cervical lesions is stronger. The signal intensity of the cervical lesion is considerably higher than that of normal tissue in the DMAP images. All these results indicate that PAI can achieve fast scanning over the whole cervix for the diagnosis of CC. The combination of photoacoustic and ultrasound imaging has been demonstrated well in terms of tissue morphology and contrast. However, for some molecular cells, PAI tends to be achieved by combining specific targeted agents due to the lack of adequate contrast. A. Agarwal et al. studied cancer cell targeting using antibody-conjugated gold nanorods for high-contrast photoacoustic imaging [67]. Prostate cancer detection could be achieved via the combination of ultrasound and photoacoustic imaging based on the contrast agent. Compared to nontargeted background tissue, combining targeted tissue with gold nanorods produces high contrast. A clear anatomical view is produced by combining photoacoustic-based cell-specific targeting with ultrasound imaging, as shown in Figure 7B. The combined image clearly shows prostate tissue implanted with gold nanoparticle samples in the photoacoustic (red) image and background tissue morphosis in the ultrasound (grayscale) image, which presents a highly attractive prospect for the early detection of prostate cancer. The detection and treatment monitoring of prostate cancer can be achieved via photoacoustic endoscopy. S. Tang et al. optimized the configuration of the PAE system through theoretical calculations and simulations [121]. The simulation proved that a combination of a transurethral laser and a transrectal ultrasound transducer is an optimal configuration, which could be used to estimate the drug concentration in vivo and guide laser-induced prostate therapy.

Alexander Dima et al. developed a compact handheld system of Photoacoustic Tomography (MSOT) that was used to image thyroid anatomy [122]. The curved ultrasound array geometry instead of conventional linear arrays was adopted to provide high-fidelity data collection and fit closely into the human neck. Figure 7C-a shows the location of the imaging plane and the anatomical structure of the thyroid. Figure 7C-a,C-b are photoacoustic images of thyroid lobes, which can identify the carotid artery, trachea, and thyroid lobe. Figure 7C-c is the corresponding ultrasound image in grayscale, which provides consistent results when comparing tissue anatomy with photoacoustic results. The directional power Doppler signals superimposed in color within the yellow rhombus verify the various sizes and flow directions of the vessel in the thyroid. Its high sensitivity regarding the finer vascular networks assists in diagnosing malignant thyroid nodules [123].

### 3.4. Animal Organ Imaging

**Figure 8:**
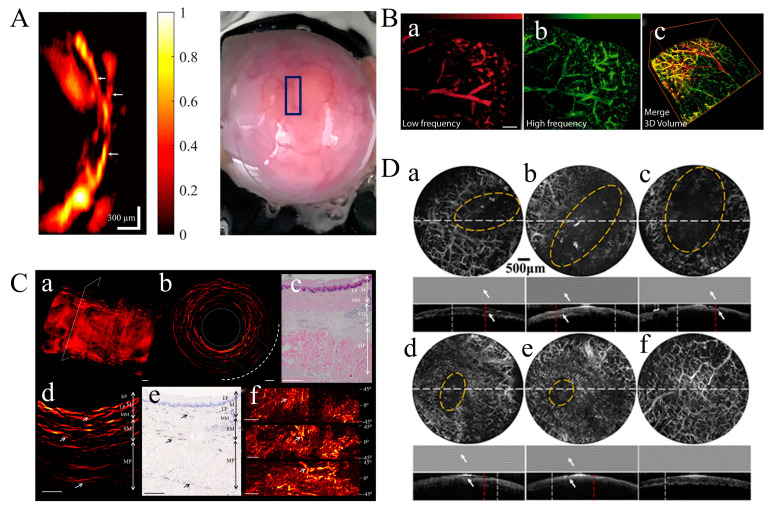
(**A**) PAM image of the vascular on a porcine ovary and corresponding photograph of the porcine ovary. The normalized PA amplitude is indicated by color bar (reproduced from Ref. [108]). (**B**) PA image and fused 3D volume overlay (**c**) of vasculature system from freshly excised liver tissue using low-frequency (10–30 MHz) (**a**) and high-frequency (30–90 MHz) (**b**) detection (reproduced from Ref. [10]). (**C**) Imaging of pig esophagus ex vivo. (**a**) Volumetric PA image of esophagus sample. (**b**) PA image of the region of cross-sectional esophagus wall in the dotted box corresponding to figure (**a**). (**c**) Histological image with the different layers of esophagus wall. EP: epithelium; M, mucosa; LP, lamina propria; MM, muscularis mucosa; MP, muscularis propria; SM, submucosa. (**d**) PA image of the different layers in the dotted box corresponding to figure (**b**). (**e**) Vasculature of the different esophageal layers revealed by anti-CD31 immunostaining. (**f**)The stained histological image of the different esophageal layers and corresponding to PA image of different layers. (reproduced from Ref. [129]). (**D**) The microvascular distribution of lower lip during the healing of an ulcer wound. Row 1 in (**a**–**f**) presents ORPAM MAP images of ulcer wound. ORPAM B-scans of lip along the dashed white lines in row 1 are shown in row 2 (**a**–**f**). OCT B-scans are shown in row 3 (**a**–**f**) (reproduced from Ref. [131]).

Mohsen Erfanzadeh proposed fast and low-cost photoacoustic microscopy based on a laser scanning laser diode that was used to image an ex vivo porcine ovary. Figure 8A shows the PAM image of the ex vivo porcine ovary and its photograph. Clear branches of blood vessels are clearly shown in the PAM image corresponding to the blue rectangle region. The ~21 μm resolution for resolving such vessels on the porcine ovary is displayed in this system, which indicates 50–65 μm Full Width at Half-Maximum (FWHM) vessel branches. The potential sensitivity to arterioles, venules, and angiogenic sprouting clusters provides significant help for the imaging and classification of ovarian cancer and the clinical study of ovarian cancer [126,127,128]. Ferdinand Knieling et al. demonstrated the capability of Raster-Scanning OA Mesoscopy (RSOM) in vivo gastrointestinal imaging [10]. A custom-made spherically focused detector with a center frequency of 50 MHz and a bandwidth of 10–90 MHz was used to detect the signals divided into two frequency bands, 10–30 MHz (low) and 30–90 MHz (high). The vasculature of the freshly excised liver is presented, which indicates big vessels (red) in the low-frequency signals and small vessels (green) in the high-frequency signals, as shown in Figure 8B-a,B-b, respectively. Figure 8B-c shows a merged 3D volume image. 

Monitoring the early pathological changes and detecting the severity of esophageal cancer supports the treatment and survival of patients [123,124]. Hailong He et al. proposed a novel Capsule Optoacoustic Endoscopy (COE) system to image a volumetric vascular network in swine esophageal lining [129,130]. The volumetric optoacoustic image of the ex vivo pig esophagus is shown in Figure 8C-a with a wavelength of 532 nm due to the optimal absorption spectrum of hemoglobin. Figure 8C-b shows a cross-sectional Radial Maximum Intensity Projection (RMIP) corresponding to the dashed line in Figure 8C-a. Figure 8C-d is the enlarged area of the rectangle in Figure 8C-b, which highlights the network of blood vessels within the esophageal area. Histology sections of the region enclosed by the rectangle stained with two different dyes are shown in Figure 8C-c,C-e, respectively. Figure 8C-c displays the layered morphology of the esophageal wall, including the mucosa (M), submucosa (SM), and puscularis propria (MP) [127]. The arrows in Figure 8C-d,C-e indicate vasculature features in different esophagus layers. Figure 8C-f shows the RMIPs of the volumetric region along the longitudinal direction of the esophagus corresponding to the rectangular area in Figure 8C-b. The delamination of the esophageal wall can be achieved using the vessel diameter, which was resolved down to a depth of 2 mm. The analysis of the superficial layer structure of the esophagus wall assists in the early detection and staging of esophageal cancer. 

More and more people’s health problems are plagued by oral diseases, especially oral cancers. The imaging of vasculatures and structures in the human oral cavity is crucial for learning more about oral-related diseases. Wei Qin et al. proposed a dual-modality portable Optical Resolution Photoacoustic Microscopy (OR-PAM) and optical coherence tomography (OCT) system, which could achieve a lateral resolution of 8.1 μm (ORPAM) and 8.56 μm (OCT) and an axial resolution of 116.5 μm (ORPAM) and 6.1 μm (OCT) [131]. The vivo imaging study of a lip ulcer in a volunteer’s oral cavity was indicated to monitor the healing process in Figure 8D. The wound region of a human lip ulcer in different stages with dashed yellow circles is shown in the first row of Figure 8D-a to Figure 8D-f. The B-scans of ORPAM and OCT are shown in the second and third rows, which present the structure changes during the healing process. The wound position is indicated by white arrows. The wounds are indicated by yellow circles in ORPAM MAP images and white arrows in ORPAM B-scans. The defect in the microstructure that is hard to resolve is overcome by integrating ORPAM and OCT in this portable probe, which better visualizes both the microvasculature and the microstructure of oral tissue. Moreover, imaging based on ORPAM and OCT also achieved quantitative analyses for ulcer wounds, including the wound area and total hemoglobin. The ORPAM system has also been reported for oral imaging by Tian Jin et al. [132]. A larger field of view (FOV) and Depth of Field (DOE) are simultaneously obtained in this system.

### 3.5. Brain Imaging

**Figure 9:**
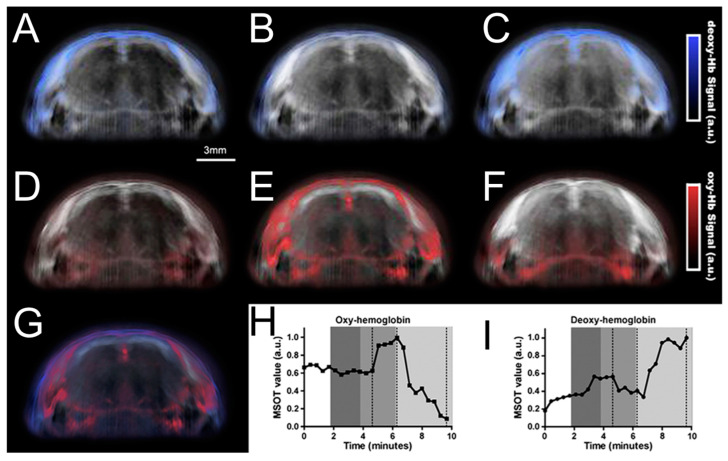
Brain blood oxygenation stimulated by carbon dioxide. (**A**–**C**) Deoxy-hemoglobin pseudocolor images from a single animal at different time points. (**D**–**F**) The darker blue represents an increase in deoxidation. The corresponding oxy-hemoglobin is in red. (**G**) shows a combination of oxy- and deoxy-hemoglobin signals corresponding to B and E. (**H**,**I**) The change in oxy- and deoxy-hemoglobin signals with time (reproduced from Ref. [133]).

The study of brain function and disease relies heavily on the analysis of imaging tissues. Imaging techniques for changes in blood volume and oxygen consumption related to brain physiology and pathology help visualize the dynamic and functional properties of the nervous system. Neal C. Burton et al. proposed a Multispectral Opto-Acoustic Tomography (MSOT) system, which achieved time-dependent detection and quantification of mouse brain parameters [133,134]. Unlike the ability of single-wavelength imaging to yield the absorption contrast of different tissue structures, multiple-wavelength illumination could process specific spectral signatures of tissue molecules, exogenously absorbing agents and nanoparticles with certain spectral signatures. Figure 9 shows the changes in oxygenation and deoxygenation in the mouse brain using multispectral image acquisition. Deoxy-hemoglobin pseudocolor images are displayed for different periods of time in Figure 9A–C. The blue concentration indicates increasing deoxygenation. Figure 9D,E show changes in the oxygenated hemoglobin concentration at the same time point. Figure 9G shows an overlap of deoxy- and oxy-hemoglobin signals corresponding to Figure 9B,E. A plot of oxy- and deoxy-hemoglobin signals is shown in Figure 9H,I. MSOT technology achieved not only cross-sectional imaging throughout the entire brain but also determined the spatial resolution of the blood volume and oxygen saturation/hypoxia, which play a crucial part in brain functional studies, disease development, and evaluation of treatment.

**Figure 10:**
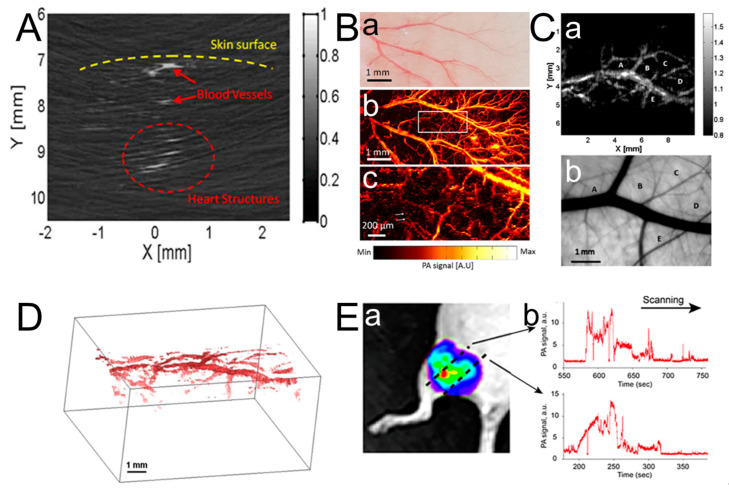
(**A**) Photoacoustic B-scan image of the beating heart in an athymic nude mouse. The preliminary image of cardiac structures is depicted, including blood vessels and the skin surface (reproduced from Ref. [135]). (**B**) Photograph of the mouse ear showing blood vessels (**a**) and corresponding PA images (**b**,**c**). (**c**) is an enlarged region of the white line box in (**b**). Red blood cells are presented by the white arrows (reproduced from Ref. [136]). (**C**) (**a**) MAP photoacoustic image of subcutaneous blood vessels in the upper dorsal region of rat. (**b**) The photograph from the dermal side of excised skin with transmission illumination corresponding to (**a**). A–E is the area enclosed by major blood vessels (reproduced from Ref. [138]). (**D**) In vivo 3D photoacoustic images of the upper dorsal region of rat (reproduced from Ref. [138]). (**E**) (**a**) The intravital whole-body imaging of mice after inoculating the tumor for a period of time. (**b**) The diagram of photoacoustic signals, which is obtained by scanning along two black dashed lines (reproduced from Ref. [139]).

The genes of mice have a high similarity with those of humans, and their genetic genes are relatively stable, so a study of various tissues and organs of mice was carried out in photoacoustic imaging for future clinical applications in the human body. Studies of cardiovascular disease often require high-frame-rate imaging modalities due to rapid heart rates. R.J. Zemp presented a unique real-time photoacoustic microscopy system for imaging beating hearts in young athymic nude mice in vivo [135]. A high-repetition-rate laser and a high-frequency ultrasound array transducer were adapted in this system to improve the imaging speed and imaging resolution. Figure 10A shows the imaging of the beating heart of the same mouse. B-scan photoacoustic imaging with a single optical wavelength of 578 nm and an isosbestic point provides visualization of optical absorption structures up to depths up to ~3–4 mm, which makes their motion evident with cardiac and respiratory cycles. The dramatic changes in vascular visibility during respiration are due to venule dilation during respiration-induced changes in the intrathoracic pressure, which may have important implications for studying venous return and diastolic function. The oxygen saturation of tissue is related to tissue behavior, which provides important solutions for studying the developmental causes and consequences of ventricular septal defects in which oxygenated and deoxygenated blood mixes during the cardiac cycle. Jin Young Kim developed Optical-Resolution Photoacoustic Microscopy (OR-PAM) that achieved a fast imaging speed (at five B-scan images per second) and high SNRs [136,137]. PA imaging of microvasculatures in a mouse ear was successfully achieved. The axial and lateral resolutions were quantified as 27.7 and 3.6 μm. Figure 10B shows the photograph of the mouse ear and the corresponding in vivo PA Maximum Amplitude Projection (MAP) image. The large blood vessels, small capillaries, and individual red blood cells (RBCs) along the capillaries were all clearly visualized. During in vivo non-invasive imaging of mice, OR-PAM achieved good imaging performance in terms of the SNR, resolution, and scanning range. Liang Song et al. proposed an in vivo dark-field reflection-mode photoacoustic microscopy system, which achieved 3D imaging at a speed of 166 B-scan frames per second [138]. The quantified lateral and axial resolutions were 70 and 25 μm, respectively. The noise signals from superficial layers of the skin could be suppressed by the dark-field laser pulse illumination. An in vivo MAP photoacoustic image of the subcutaneous vasculature and ex vivo transmission optical microscopic image is shown in Figure 10C. The vascular distribution has a good match between the two images. The diameter of blood vessels varied from 80 to 700 μm. The volumetric image of subcutaneous vasculature in rats is shown in Figure 10D. Fast 3D photoacoustic microscopy with a 30 MHz ultrasound linear-array transducer achieved real-time B-scan imaging at 50 Hz and 3D imaging at 1 Hz. Moreover, the exchange of oxygen and nutrients between blood and tissues in capillaries is essential for basic physiological studies. 

### 3.6. Hematologic Imaging

The spread of cancer cells between organs tends to be accompanied by the occurrence of circulating tumor cells. The detection of circulating tumor cells could be helpful for diagnosing cancer early and preventing its metastasis. Dmitry A. Nedosekin et al. introduce a novel cerebrospinal fluid (CSF) based on in vivo Photoacoustic Flow Cytometry (PAFC) and ex vivo photothermal scanning cytometry, which molecularly detected in vivo circulating tumor cells (CTCs) before the development of breast cancer brain metastasis [139]. Compared to other detection methods, Photoacoustic Flow Cytometry (PAFC) is sensitive to light scattering, which has advantages in the diagnosis of diseases at the single-cell level in vivo, such as the detection of CTCs and leukocytes in blood and lymph flows as well as bacteria in circulating blood [140,141]. Figure 10 E-a shows photoacoustic signals of primary tumors injected into mice, which are detected by two kinds of wavelength of 670 nm and 820 nm. The primary tumor is identified by the bright spot in the bioluminescent image in Figure 10E-a, which shows higher signal amplitudes in Figure 10E-b. In their study, the real-time production of CTCs in CSF could be estimated, which enhances the understanding of CTCs in the development of cancer metastasis. Moreover, a magnetic capture system combining photoacoustic detection was developed by Ekaterina I. Galanzha et al., which achieves the capture and detection of circulating tumor cells [142]. Compared to the PAFC system, the system integrates in vivo multiplex targeting, magnetic enrichment, signal amplification, and multicolor recognition so that the sensitivity and specificity are improved. Characteristics of the studies covered in this review is shown in Table 2.

## 4. Conclusions and Outlook

Imaging technology based on the photoacoustic effect has rapidly advanced biomedical applications in recent years. This extensive research has mainly been promoted by a sea of unmet biological and medical needs and provides non-invasive, label-free, and non-ionizing imaging. It overcomes the defect of pure optical scattering and provides a higher resolution and optical absorption contrast. The combination of PAI with existing imaging techniques (multimodality imaging) or the delivery of therapeutics potentially offers wider applications. 

Although PAI technology has been utilized in more clinical applications, there are still many challenging problems to be studied in depth in biomedical imaging science, including imaging instrumentation, superior reconstruction algorithms, spectral processing schemes, detection sensitivity, and imaging performance [24]. The photoacoustic probe and the geometry of the transducer array significantly influence the detection sensitivity. The size of the photoacoustic probe should be scaled down to <1 mm in diameter to be suitable for intravesicular and intravascular PAI [143]. A more universal geometry array of transducers should be developed for detection with a limited view and improved sensitivity. Moreover, the drawback of the PAI system based on the laser diode is that the non-rotationally symmetric beam is more difficult to collimate or focus into a diffraction-limited spot [99]. The reconstructed imaging generally contains artifacts due to non-ideal properties of biological tissue. Inaccurate assumptions regarding the speed of sound and acoustic attenuation deeply affect the reconstructed image accuracy [117]. The penetration depth, one of the imaging performance indicators, can be achieved on a smaller range of several centimeters so that deeper tissue information cannot be obtained. The attenuation of light influence in optically opaque tissue creates further limitations. The compensated method increases the amount of the deposited laser energies within the range of the Maximum Permissible Exposure (MEP). As a result, the imaging depth depends on the detectable pressure changes produced in the region of high light fluence. In addition, due to the dependence of the spatial resolution on the penetration depth, PA signals of microscopic lesions (a few mm) in deeper tissue are difficult to detect. 

The ability to obtain functional and molecular information from tissue meets the requirements of multiple clinical areas over the currently available imaging techniques. A wide variety of imaging scanners and devices of light excitation have been rapidly developed in numerous laboratories around the globe for experimental and clinical applications. The need for PAI equipment will emphasize more efficient and affordable laser technology and miniaturized and sensitive ultrasound detection approaches. Enhancing imaging performance will focus primarily on developing new parallel computation platforms and advanced reconstructed algorithms for data processing. The continuous introduction of new contrast agents, from nanoparticles and organic dyes to targeted agents and genetically encoded markers, has optimized the optical properties and biological characteristics. This further facilitated wide applications and rapid developments. Future PAI techniques will cover broader and deeper research of modern biology in preclinical applications in cancer, cardiovascular diseases, neuroimaging, ophthalmology, immunology, diabetes and obesity, cell trafficking, and many other biological functions [144]. Comprehensive improvements in the PAI system will continue to drive technological change in biomedical research as an important frontier tool.

## Figures and Tables

**Table 1:** Achieved imaging performance.

Modality	Spatial Resolution (L: Lateral Resolution, A: Axial Resolution)	Penetration Depth	Validation
PAE	L: 421 μm, A: 69 μm	1.5 mm	Esophagus wall imaging of a pig
PAM	L: 70 μm, A: 25 μm	3 mm	Subcutaneous vasculature in rats
PAT	L: 140 μm, A: 40 μm	15 mm (40 Mhz)	Subcutaneous vasculature in human forearm
PAT	L: 30 μm, A: 7 μm	5 mm	Drosophila fly and drosophila ex vivo
OR-PAM	L: 6.2 μm, A: 27 μm	3.2 mm	Fresh chicken breast tissue
AR-PAE	L: 18 μm, A: 4 μm	5 mm	Phantom
PAM	L: 100 μm, A: 25 μm	3 mm	Microvessels in a rat

**Table 2:** Characteristics of the studies covered in this review.

Research		Characteristics	Reference
Application	Dermatologic imaging and joint imaging	Advantages of PAI for imaging skin diseases.	[100,101,102,103,104,105,106,107,108,109,110,111,112,113]
	Breast tumor imaging and vascular disease detection	Advantages of high contrast and high spatial resolution of PA imaging for breast cancer diagnosis.	[114,115,116,117]
	Cervical cancer	Optimization of PAI for cervical cancer diagnosis.	[67,71,118,119,120,121,122,123,124,125]
	Animal organ imaging	Effective application of PAI on animal organs.	[10,108,126,127,128,129,130,131,132]
	Brain imaging	PAI is used in brain imaging to assist the research of image analysis.	[133,134,135,136,137,138]
	Hematologic imaging	Detection of circulating tumor cells by PAI.	[135,136,138,139,140,141,142]

## Data Availability

No data were generated or used during this study.
